# A theoretical framework for the regulation of Shh morphogen-controlled gene expression

**DOI:** 10.1242/dev.112573

**Published:** 2014-10

**Authors:** Michael Cohen, Karen M. Page, Ruben Perez-Carrasco, Chris P. Barnes, James Briscoe

**Affiliations:** 1 MRC-National Institute for Medical Research, The Ridgeway, Mill Hill, London NW7 1AA, UK; 2 Department of Mathematics and CoMPLEX, University College London, Gower Street, London WC1E 6BT, UK; 3 Department of Cell and Developmental Biology and Department of Genetics, Evolution and Environment, University College London, Gower Street, London WC1E 6BT, UK

**Keywords:** Approximate Bayesian computation, Enhancer, Gene regulation, Gli, Morphogen patterning, Shh, Mathematical modelling, Transcriptional networks

## Abstract

How morphogen gradients govern the pattern of gene expression in developing tissues is not well understood. Here, we describe a statistical thermodynamic model of gene regulation that combines the activity of a morphogen with the transcriptional network it controls. Using Sonic hedgehog (Shh) patterning of the ventral neural tube as an example, we show that the framework can be used together with the principled parameter selection technique of approximate Bayesian computation to obtain a dynamical model that accurately predicts tissue patterning. The analysis indicates that, for each target gene regulated by Gli, which is the transcriptional effector of Shh signalling, there is a neutral point in the gradient, either side of which altering the Gli binding affinity has opposite effects on gene expression. This explains recent counterintuitive experimental observations. The approach is broadly applicable and provides a unifying framework to explain the temporospatial pattern of morphogen-regulated gene expression.

## INTRODUCTION

In many developing tissues, pattern formation depends on the differential regulation of gene expression by morphogen gradients (Kicheva et al., 2012; Rogers and Schier, 2011). To understand tissue patterning, predictive mechanistic models of morphogen-dependent gene regulation are needed.

**Fig. 1.**
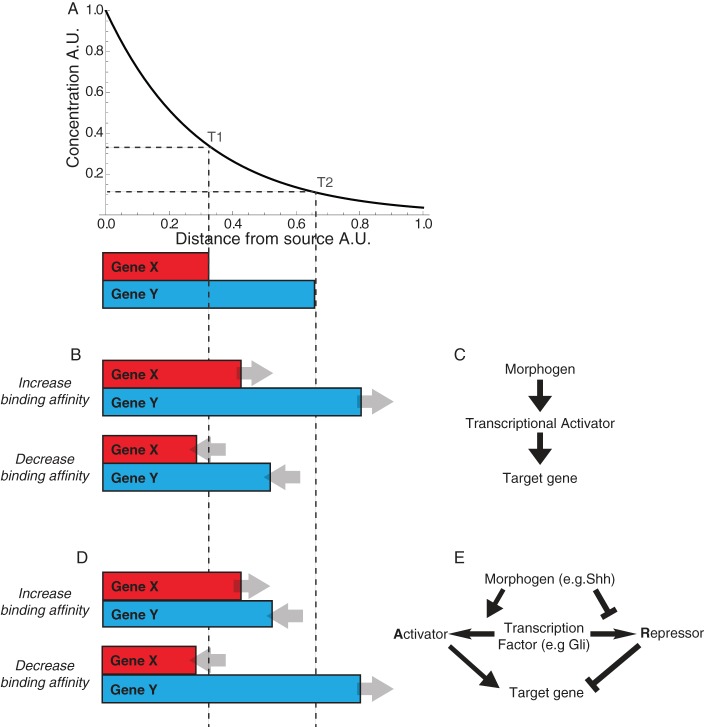
**Morphogen regulation of gene expression by an activator/repressor effector.** (A) A morphogen gradient determines the range of expression of two genes: X and Y. The boundaries of X and Y are defined by the concentration thresholds T1 and T2. (B) The affinity threshold model predicts that genes expressed further from the morphogen source will have higher binding affinities (gene Y) compared with genes expressed over a shorter range (gene X). For both genes, in this model, increasing the binding affinity will increase the ranges of gene expression, whereas decreasing binding affinity will reduce them. (C) The affinity threshold model is viable in a simple case in which a morphogen directly activates (or acts as) a transcriptional effector that acts solely as an activator (or, equivalently, in which the morphogen directly represses a transcriptional repressor). (D) In contrast to B, observations from Hedgehog (Hh) patterning systems indicate that binding affinity for the morphogen effector is higher for genes that are expressed closer to the morphogen source (gene X) than genes expressed at a greater range (gene Y). In experiments altering the binding affinity of the transcriptional effector, some genes behave in line with the affinity threshold model (gene X), whereas others behave in the opposite way (gene Y), decreasing their range in response to an increase in binding affinity and increasing their range in response to a decrease (Oosterveen et al., 2012). (E) The Hh pathway is an example of a signalling pathway that culminates in a bifunctional transcriptional effector. Morphogen signalling increases the concentration of the activator form and decreases the concentration of the repressor form of the effector. Both activator and repressor act on target genes through the same binding sites. A. U., arbitrary units.

A simple model postulates that a morphogen activates target genes by directly activating a latent transcriptional activator (Driever et al., 1989). The increase in the activity of the morphogen-regulated transcription factor (MR-TF) results in increased binding to its cis-regulatory elements in target genes, thereby increasing the probability of target gene expression. This model predicts that genes with few or low-affinity binding sites for the MR-TF would only be induced close to the morphogen source, where MR-TF concentration is at its highest. Conversely, genes expressed at a greater distance would have more or higher affinity binding sites. This relationship has been termed the affinity threshold model (Rushlow and Shvartsman, 2012) (Fig. 1A-C).

Support for the affinity threshold model came from experiments dissecting the molecular mechanism of gene regulation in the early *Drosophila* embryo (Driever et al., 1989; Jiang et al., 1993, 1992). Subsequent observations, however, were inconsistent with the model (Ochoa-Espinosa et al., 2005; reviewed by Cohen et al., 2013). Moreover, binding sites in the enhancers of target genes for transcription factors (TFs) other than the MR-TF have been shown to influence gene expression (Hong et al., 2008; Ip et al., 1992; Jaeger, 2004; Kanodia et al., 2012; Porcher and Dostatni, 2010). These additional TFs can be expressed uniformly throughout the tissue or controlled by graded signals. This led to the suggestion that the response to a morphogen is the product of the structure of the transcriptional network comprising the target genes. (Balaskas et al., 2012; Hong et al., 2008; Jaeger, 2004; Kanodia et al., 2012; Manu et al., 2009; Porcher and Dostatni, 2010).

It is also unclear how the affinity threshold model would apply to MR-TFs that are bifunctional, acting as transcriptional repressors in the absence of morphogen and as activators otherwise (Barolo, 2002). If activator and repressor bind similarly to the same DNA binding sites, changing binding affinity would affect the probability of bound repressor or activator equally. In *Drosophila*, Hedgehog regulates the activity of the bifunctional effector Ci (Basler and Struhl, 1994). In the wing disc, its target gene *dpp* is induced at low Hedgehog concentrations and contains three low-affinity Ci binding sites (Jiang and Hui, 2008), whereas *ptc* is induced by high Hedgehog levels and contains high-affinity Ci binding sites (Parker et al., 2011). Besides this counterintuitive allocation of binding site affinity, experimentally increasing the Ci binding site affinity in the *dpp* enhancer decreased its range of expression, which contradicts the affinity threshold model (Parker et al., 2011). To explain this, a differential cooperativity model was proposed, in which there is self-cooperative binding of the repressor but not activator isoform of Ci. The difference in cooperativity would mean that target genes with higher binding site affinity are more sensitive to repressor than activator.

In vertebrates, Sonic hedgehog (Shh), acting through Gli proteins (the Ci orthologues) (Jiang and Hui, 2008), patterns the neural tube by inducing the nested expression of a set of TFs within neural progenitors (Alaynick et al., 2011). The pan-neural transcriptional activator Sox2 provides neural specificity to these Shh target genes (Bailey et al., 2006; Graham et al., 2003; Oosterveen et al., 2013; Pevny and Placzek, 2005). Both the activator and repressor form of Gli bind to the same consensus Gli binding sites (GBS) in the genome (Hallikas et al., 2006; Muller and Basler, 2000; Peterson et al., 2012; Vokes et al., 2007, 2008) and analysis of GBSs within enhancers of Shh target genes failed to find a positive correlation between binding site affinity and range of gene induction (Oosterveen et al., 2012; Peterson et al., 2012) (Fig. 1D,E).

Differential cooperativity mechanisms (Parker et al., 2011) are unlikely to explain the behaviour of Shh target genes in the neural tube. There is no evidence that Gli repressor (GliR) isoforms bind more cooperatively than Gli activator (GliA) isoforms (Nguyen, 2005). In addition, many Shh target enhancers in the neural tube have a single GBS, thus precluding cooperative binding (Peterson et al., 2012; Vokes et al., 2007). An alternative is that the dynamics of the transcriptional network, which is composed of Gli proteins, uniformly expressed TFs and TFs downstream of Shh signalling, explains the spatial pattern of gene expression in the neural tube (Balaskas et al., 2012).

Here, we develop a mathematical model to test this idea. This reveals a mechanism that relies on the enhancer integrating input from the MR-TF with the other transcriptional inputs. We first demonstrate the logic of this model in the context of a single gene regulated solely by an MR-TF. We then extend the model to include other TFs, using patterning in the ventral neural tube as an example (Balaskas et al., 2012). The model is parameterised using a Bayesian computational technique (Liepe et al., 2010) and reproduces the experimentally observed expression pattern upon changes in Gli binding affinity. We suggest that this mechanism provides a general strategy for the regulation of morphogen-controlled gene responses.

## RESULTS

### A thermodynamic ensemble model of gene regulation

To explore the function of a bifunctional MR-TF we describe transcriptional regulation using a statistical thermodynamic formulation (Bolouri and Davidson, 2003; Shea and Ackers, 1985; Sherman and Cohen, 2012). In this approach, the thermodynamic states of the cis-regulatory system represent all possible bound configurations of DNA to polymerase and a specified set of TFs. Transcriptionally active states are those in which polymerase is bound at the promoter; inactive states have no polymerase bound. The probability of gene expression, *ϕ*, is defined by the ratio of probabilities of all transcriptionally active states to all possible states.

The statistical weight associated with each state is derived from the Gibbs free energy of each binding interaction (Shea and Ackers, 1985). In equilibrium, these weights can be represented as the product of the concentration, [X], of each binding species multiplied by the equilibrium binding constants, K, associated with that interaction (Sherman and Cohen, 2012), thereby providing a direct molecular correlate for the model. The level of cooperative binding between activator or repressor with polymerase is defined by the factor *c*. Thus *c*>1 for an activator and 0≤*c*<1 for a repressor. Cooperative binding among TFs at different sites (including self-cooperative binding of the same species) can be characterised in the same way. The weighted terms for all the states are proportional to the effective concentration of DNA, which therefore can be factored out of the equation. Hence, *ϕ* can be defined as:
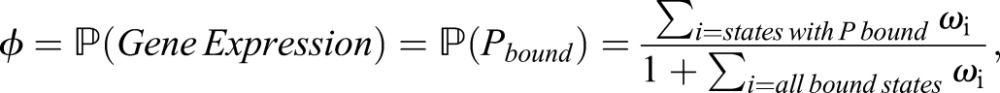
with
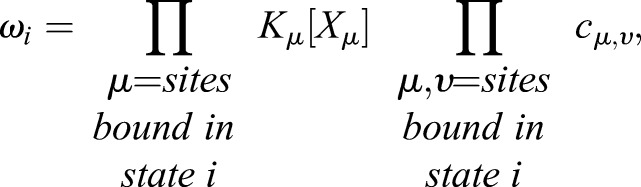
where denotes probability, P polymerase, *i* denotes each state, *μ* each of the bound DNA sites comprising that state, [*X_μ_*] the concentration of the species bound at site *μ*, *K_μ_* the binding affinity of that interaction, and c*_μ_*_,*υ*_ the level of binding cooperativity between two occupied sites *μ* and *υ*.

This formulation can recapitulate the more familiar Hill function description (based on Michaelis–Menten kinetics) of transcriptional regulation if binding of a single species is considered (see Eq. 2, representing just the binding of polymerase). However, as we discuss below in the context of the combinatorial effect of multiple TFs, the basal levels of transcription in the presence of ubiquitous regulators cannot be ignored. These effects are typically approximated by multiplying or summing separate Hill functions; however, in contrast to the thermodynamic formalism, this does not accurately represent multiple binding at different sites (Sherman and Cohen, 2012).

### A model of Gli binding at a single GBS

**Fig. 2.**
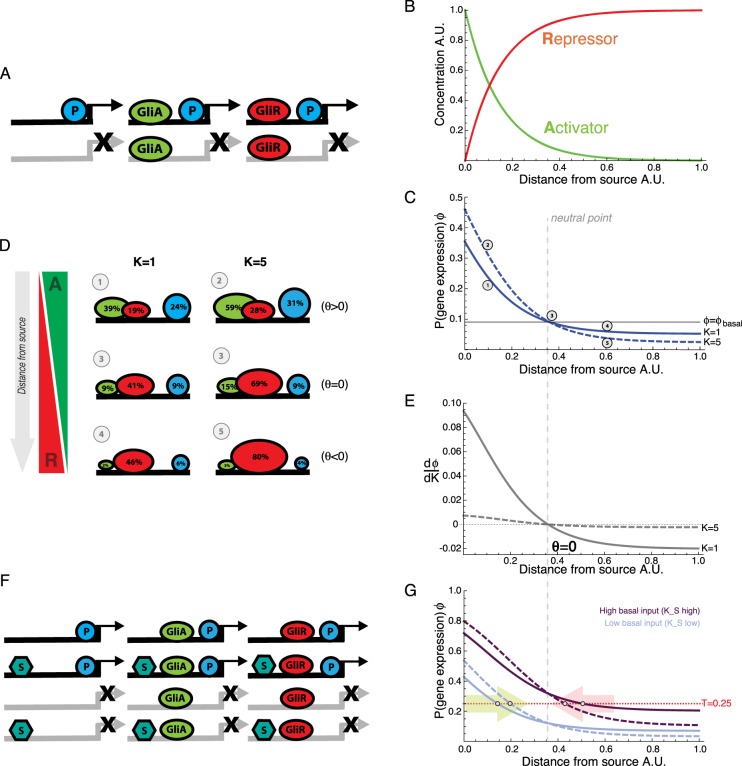
**Model of a bifunctional morphogen effector explains how binding affinity affects gene responses.** (A) All possible DNA binding configurations for the model described by Eq. 1. An enhancer composed of a single MR-TF (Gli) binding site regulates the recruitment of RNA polymerase (P) to the promoter. GliA or GliR binds at the enhancer in a mutually exclusive manner and increases or decreases (respectively) the probability of P binding at the promoter of the target gene. All transcriptionally active configurations (those with bound P) are shown with black arrows; inactive configurations are shown with crossed grey arrows. (B) For simulations, opposing gradients of activator [A] and repressor [R], which are representative of the bifunctional morphogen effectors (Gli/Ci) in a Hh morphogen gradient, were used. These were defined such that A=e^−x/0.15^, R=1−A. (C) Spatial gradients of the activator [A] and repressor [R] isoforms of Gli from (B) applied to a model of gene expression with a single Gli binding site (GBS) (Eq. 1). The probability of gene expression, *ϕ*, decreases as a function of distance from the source for target genes containing a low-affinity site (solid blue line, K=1) or a high-affinity site (dashed blue, line K=5). Close to the source there is an increase in gene expression when the binding affinity, K, for Gli is increased (compare positions 1 and 2). Far from the source there is a decrease in gene expression when binding affinity is increased (compare positions 4 and 5). The solid grey line indicates the basal level of gene expression and the dashed line the neutral point in the gradient (position 3). Remaining parameters: [P]=0.1, K_P_=0.5, c_AP_=10, c_RP_=0.1. (D) The percentage occupancy for GliA (green oval) and GliR (red oval) at the GBS (derived from Eq. 3 and Eq. 4) and P (blue circle) at the promoter (derived from Eq. 1) for different positions in the gradient depicted in the simulations in C (grey circles 1-5 located at *x*=0.1, *x*=0.35, *x*=0.6 A.U.) with different values of Gli binding affinity (K=1 and K=5) as indicated. (E) The rate of change of gene expression probability with respect to Gli binding affinity *dφ*/*dK* (Eq. 5) shown for the two cases illustrated in C. The neutral point occurs at θ=0, where the rate of change is equal to zero. θ (Eq. 6) is determined only by the concentrations of GliA and GliR and the strength of their effect on P binding (c_AP_ and c_RP_). (F) All DNA binding configurations for the model described by Eq. 7. A ubiquitously expressed TF, S, binds to the enhancer and increases the probability of P recruitment, independently of GliA and GliR binding. (G) Gene expression for two MR-TF-controlled genes, A and B (blue and purple curves), that have different basal levels of expression (Eq. 7). The blue curves (K_S_=0.1) have relatively low basal expression; the purple curves (K_S_=10) have a higher basal level of expression. Remaining parameters: [P]=0.1, K_P_=1, c_AP_=10, c_RP_=0.1. A threshold probability for gene activation is defined as T=0.25 (red line). An increase in GBS affinity (from K=1 solid lines, to K=5 dashed lines) increases the range of expression (above the threshold) of gene A (as indicated by the green arrow). Conversely, the range of expression of gene B is contracted towards the source (red arrow) when affinity is increased. For both genes, the neutral point (grey dashed line), at which the probability of gene expression does not change when K is altered, occurs at the basal level and is determined by the position in the gradient where θ=0 (as shown in E).

Here, we will consider a gene regulated by Shh-Gli signalling. However, the conclusions generalise to any morphogen pathway transduced by bifunctional transcriptional effectors. Using the framework described above we can define *ϕ* for the simplest model of interest in this study, in which the morphogen activator GliA (A), and repressor GliR (R), bind to a single DNA binding site (GBS). Activation (or repression) arises through their cooperative (or inhibitory) binding with polymerase. We illustrate the different bound configurations of DNA schematically in Fig. 2A. The gene expression probability is given by:1
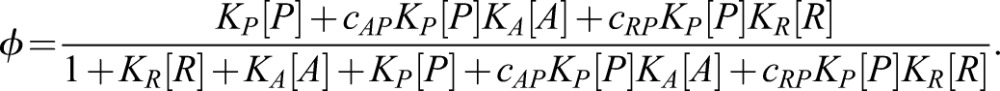

The numerator in this function represents all the transcriptional states in which polymerase is bound and transcription can be active: polymerase on its own, activator and polymerase bound, or repressor and polymerase bound. The denominator includes the active and the additional inactive states: free DNA (=1), and activator or repressor bound in the absence of polymerase. The constants K_P_, K_A_ and K_R_ represent the binding affinities of polymerase, activator and repressor, respectively. The cooperative terms c_AP_>1 and c_AR_<1 represent activation and repression, respectively.

It is worth noting that in the absence of any TFs ([A]=[R]=0) this function reduces to:2
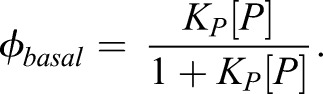
This represents the basal probability of gene expression without activation or repression.

If GliA and GliR have equal binding affinities for the GBS, then *K_A_=K_R_≡K*. To illustrate the behaviour, we define gradients of GliA and GliR that represent the transduction of an Shh gradient (Fig. 2B). The solid blue line in Fig. 2C represents the probability of gene expression across this gradient, as determined by Eq. 1, for K=1 and assuming A acts as a strong activator (c_AP_=10) and R a strong repressor (c_RP_=0.1). The horizontal grey line in Fig. 2C represents basal gene expression.

Using the thermodynamic model, the probability of occupancy of the GBS by either GliA or GliR can be determined:3
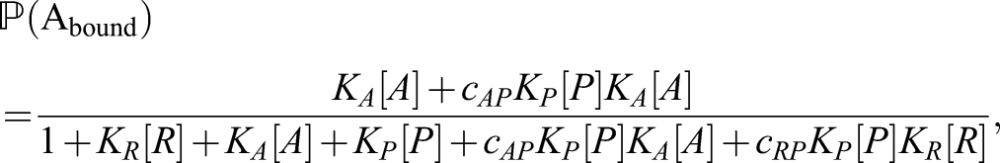
4
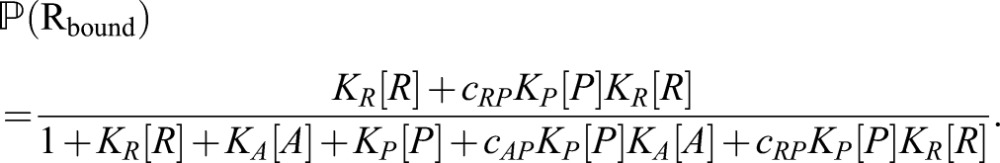


These functions are illustrated in Fig. 2D (see first column referring to positions 1, 3 and 4 in Fig. 2C). As expected, higher polymerase occupancy (equivalent to the probability of gene expression, *ϕ*) is associated with higher occupancy of GliA and lower occupancy of GliR.

### Predicting the relationship between Gli binding affinity and gene expression

We asked how *ϕ*, the probability of gene expression, changes when GBS binding affinity, K, for GliA and GliR is changed. In the affinity threshold model, an increase in binding site affinity is expected to increase the probability of expression, resulting in an increased range of gene expression. In the thermodynamic model (Eq. 1), *ϕ* varies with respect to K as:5
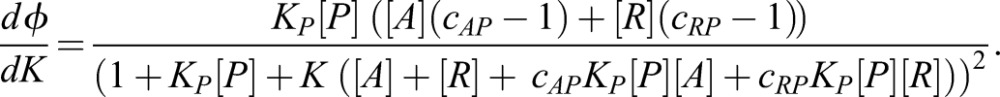


Because the denominator in this equation is always positive, the sign of the function depends only on the sign of *θ*, where *θ* is the part of the numerator that can be either positive or negative:6
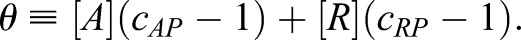


For an increase in binding affinity, K, to result in an increase in gene expression probability, *ϕ*, requires *dφ*/*dk*>0. This will occur when *θ*>0. Conversely, if *θ*<0 then *dφ*/*dk*<0, and therefore *ϕ* will decrease when the binding affinity is increased.

As *θ* depends only on the concentration of Gli isoforms and the strength of activation or inhibition, *θ* can be viewed as a function of position within the morphogen gradient (independent of K, K_P_ and [P]). For the case described, where A is an activator (c_AP_>1) and R is a repressor (c_RP_<1), *θ*, and therefore *dφ/dk*, will be positive only in regions of the gradient close to the source where A is high and R is low. Conversely, *dφ/dk* will be negative further from the source, where R is high and A is low (Fig. 2E).

Simulations confirm that gene expression probability changes when Gli binding affinity is altered (Fig. 2C; supplementary material Fig. S1). As predicted analytically, an increase in binding affinity increases the probability in the regions closer to the source (*dφ*/*dk*>0) and decreases probability in regions further from the source (*dφ*/*dk<*0). Moreover, at the position in the gradient where *θ*=0 and hence *dφ*/*dk*=0, no change will occur when K is increased. If we insert this condition back into Eq. 1 we obtain *ϕ*=(*K_P_*[*P*])/(1+*K_P_*[*P*]), which is the basal level (see Eq. 2) in the absence of any TF binding. Thus, there is a ‘neutral point’ in the gradient at which gene expression is fixed at basal levels (solid grey line in Fig. 2C). Close to the source, where gene expression is above basal levels, an increase in GBS binding affinity causes an increase in the probability of gene expression. Further from the neutral point, where gene expression is below basal levels, increasing binding affinity decreases the probability of gene expression. The position of the neutral point is determined only by the concentrations of GliA and GliR and the strength of their cooperative binding with polymerase (Eq. 6) and is independent of the basal level of gene expression.

The effect of binding affinity on enhancer occupancy is illustrated in Fig. 2D. Increasing the affinity of both GliA and GliR binding at all positions in the gradient (compare left and right columns) results in an overall increase in the probability that a GBS will be bound relative to the unbound state. Close to the source of morphogen, an increase in K causes a net increase in GliA bound and hence polymerase occupancy and gene expression probability increases (compare positions 1 and 2); further from the source, a net increase in GliR bound results from higher values of K and polymerase concomitantly decreases (compare positions 4 and 5). At position 3, the neutral point, there is a balance of activation and repression and gene expression is at the basal level. This is not equivalent to having the same amount of GliA and GliR bound at the enhancer: when K is increased, both isoforms of Gli still increase their binding proportionately but there is no net change in the level of gene expression.

Implicit in the thermodynamic model (Shea and Ackers, 1985) is a reciprocity in the binding cooperativity between a TF and polymerase. In supplementary material Section 2, we review an alternative model in which polymerase does not affect the binding or unbinding of Gli. In this model, there is still a neutral point in the gradient, either side of which gene expression probability will increase or decrease when binding affinity is altered. However, the neutral point changes position within the gradient when the basal level of expression changes (supplementary material Fig. S2). For both models it is possible to represent the gene expression probability in a form that is linearly proportional to the relative abundance of GliA to GliR (see supplementary material Section 3).

A second assumption of the model is that TF binding to DNA is fast relative to changes in TF concentration. Hence, the probability of gene expression given by the model is the probability reached at equilibrium with respect to the TF and polymerase concentrations. To determine the dynamic response of these systems prior to steady state, a more complex model comprising the on/off binding rates for each of the different factors would be required.

Thus far this analysis has described models consisting of a single binding site with equal affinity for the activator and repressor. A qualitatively similar result is obtained if the number of binding sites is altered instead of their affinity (see supplementary material Section 1 and Fig. S1B). More complex regulatory systems, in which there is differential binding affinity or cooperativity among multiple binding sites, can also be applied (see supplementary material Section 4 and Fig. S3) (Parker et al., 2011). The key result still holds for these models; close to the source increasing binding affinity will increase expression probability and far from the source it will decrease. Hereafter, we apply the thermodynamic model comprising a single GBS, but the fundamental findings are consistent among these alternative models.

### Transcriptional inputs other than the morphogen can determine boundary positions

The binding of TFs other than the morphogen effector is also likely to influence the level of gene expression. Indeed, previous studies have demonstrated how the levels of binding of a spatially uniform factor to target genes in a morphogen patterning system can significantly influence their expression profiles (Kanodia et al., 2012). In the neural tube, the TF Sox2 has been suggested to provide a spatially uniform activation input into neurally expressed genes (Bailey et al., 2006; Oosterveen et al., 2012; Peterson et al., 2012). Using this as an established example, we illustrate the possible bound configurations of DNA in the case of a uniformly expressed TF and a bifunctional MR-TF in Fig. 2F. An additional uniform activator, S, is incorporated into the gene expression function using the same thermodynamic formulation as described above:7
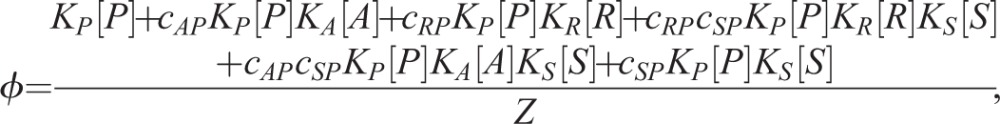
where
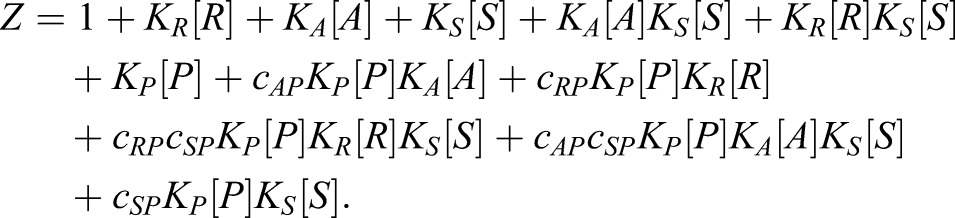


Here, S binds to DNA with an affinity K_S_ and cooperatively binds with polymerase with a factor c_SP_. The binding of S with either GliA or GliR has an additive effect on the binding energy with P, and hence a multiplicative effect on the weighted binding probability. P and S now define the basal level of gene expression, in the absence of Gli:8
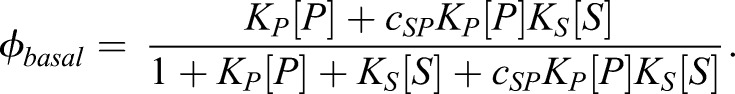


The conditions for an increase or a decrease in gene expression probability when the binding affinity of Gli (*K≡K_A_=K_R_*) is increased is still the same as previously defined in the absence of S; however, at the ‘neutral point’ (where *dφ*/*dK*=0 at the position *θ*=0) gene expression is at this new basal level.

Fig. 2G shows an example for two solutions of *ϕ*, one with K_S_=0.1 (blue), reflecting a gene with low levels of Sox2 binding, and one with K_S_=10 (purple), reflecting a gene with high levels of Sox2 binding. Increased binding affinity for S increases the basal level of gene expression. Moreover, in both cases, if the binding affinity for Gli is increased from K=1 to K=5 (solid to dashed line) then the predicted increase or decrease in the probability of gene expression is observed either side of the neutral point. Moreover, if we consider, hypothetically, that a threshold probability at which a gene is regarded as ‘on’ occurs at a fixed level (e.g. gene expression is observed at *ϕ*∼0.25) then it is easy to see how the level of basal expression will contribute to a gene's expression boundary in the gradient (note that this will not generate a sharp boundary of gene expression; we explore this in detail in the next section). If this threshold position is closer to the gradient source than the neutral point (which is defined only by the Gli gradient) then an increase in GBS affinity will cause an expansion in the range of gene expression. This result is relevant to the transgenic assays in which increasing the GBS affinity in enhancers of Shh targets expressed in ventral regions of the neural tube resulted in an expansion in the range of gene expression (Peterson et al., 2012). Conversely, for a gene that has a threshold level of expression that is further from the gradient source than the neutral point, then a contraction in expression is predicted, as observed in more dorsally expressed genes in the neural tube (Oosterveen et al., 2012). The contraction in expression of these latter genes can be explained by the repressive effect of the MR-TF. For these genes, when the morphogen concentration is below some threshold, the repressor form of the MR-TF represses target gene expression below its basal level. Hence, increasing binding affinity of the morphogen transcriptional effector increases the overall repression in regions beyond the neutral point and thereby contracts the domain of gene expression.

For a bifunctional MR-TF the position of a target gene boundary is prognostic of how it will respond to changes in the affinity for the MR-TF. Genes with boundaries close to the source (before the neutral point) are more likely to undergo an expansion in their expression when MR-TF affinity is increased, whereas gene boundaries further from the source (beyond the neutral point) are more likely to contract if affinity is increased.

### Patterning within a gene regulatory network

**Fig. 3.**
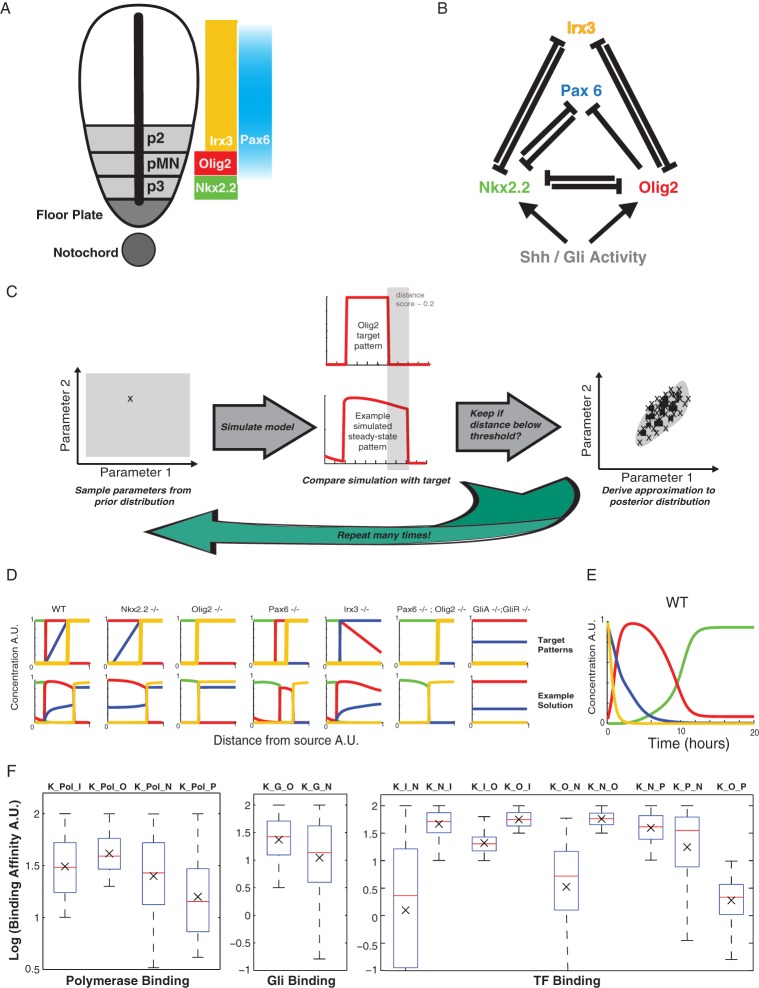
**Approximate Bayesian computation (ABC) of parameter distributions for a gene network model.** (A) In the neural tube, the notochord and floor plate provide a source of Shh. This gradient acts through Gli proteins to produce a spatial pattern of distinct progenitor domains in the ventral region (the three ventralmost domains are shown: p2, pMN, p3). Each domain is characterised by the expression of different sets of TFs; the spatial distribution of four of these (Nkx2.2, Olig2, Irx3 and Pax6) is illustrated. (B) Nkx2.2, Olig2, Irx3 and Pax6 form a cross-repressive network with the regulatory links indicated in the diagram. Pax6 and Irx3 are expressed in the absence of any Shh signal. Nkx2.2 and Olig2 are regulated by Gli activity. (C) In ABC, parameter sets are drawn from a multi-dimensional prior distribution. We illustrate the idea for a model with just two parameters. The set of parameters drawn is then used in a model simulation – in this case a pattern of each of the four genes was derived by simulating the dynamical model described in Eq. 9 to steady state. The pattern that results from the simulation is scored against a target output (see Materials and Methods for details); in this example, roughly 20% of the pattern of one of the genes (highlighted in grey) does not match the target, giving a distance score of 0.2. Parameter sets that score a sufficiently low distance are kept, the rest discarded. The posterior distribution is approximated from the distribution of the retained parameter sets. (D) The sets of target patterns for the WT and six different mutants are shown in the top row. The *x*-axis represents the ventral-dorsal position in the neural tube extending away from the morphogen source. The *y*-axis represents the protein concentration for each of the four TFs (colour-coded according to A). The bottom row shows a typical example of a simulated set of patterns using a set of parameters derived from the Bayesian analysis. The patterns represent the steady-state level of protein expression after 100 h of simulation at each position in the gradient. The parameters in this example are: α=β=2, c_AP_=10, K_G_O_=18.0, K_G_N_=37.3, K_P_N_=4.8, K_P_O_=47.8, K_P_Pax_=4.8, K_P_I_=23.4, [P]=0.8, K_N_Pax_=26.7, K_O_Pax_=1.9, K_N_O_=60.6, K_O_N_=27.1, K_Pax_N_=4.8, K_O_I_=58.8, K_N_I_=76.2, K_I_O_=28.4, K_I_N_=47.1. The mutants were simulated by setting the relevant production rate to zero. For the Gli mutants, Gli binding was set to zero. In each case, the target pattern is approximately reproduced by the simulated pattern. (E) The temporal dynamics of the model (using the example parameter set in D) at *x*=0.1 (a position that will express Nkx2.2 in the WT) are shown. The zero time point represents the start of the Shh signal (after the system has settled to a steady state). The figure shows the sequential expression of the different TFs consistent with *in vivo* data (Balaskas et al., 2012). (F) The marginal (single parameter) posterior distributions for each of the binding affinities derived in the Bayesian analysis are represented as box plots. The boxes show the median, lower and upper quartile ranges. The whiskers encompass ∼99% of the distribution. The black cross shows the mean value.

In many morphogen systems, a network of interactions between TFs that are regulated by the morphogen contributes to the spatial pattern of gene expression (Balaskas et al., 2012; Davidson, 2006; Manu et al., 2009). These morphogen-regulated transcriptional networks provide a spatially and temporally varying input into gene regulation (Bolouri and Davidson, 2003) that is distinct from the morphogen effector and any uniform modulators. To explore how a bifunctional transcriptional effector could affect patterning behaviour in this context, we applied it to the transcriptional network that functions in the ventral neural tube (Balaskas et al., 2012; Briscoe et al., 2000; Novitch et al., 2001) (Fig. 3A,B). We used the thermodynamic formulism to describe a previously documented transcriptional network (Balaskas et al., 2012; Panovska-Griffiths et al., 2013). We incorporated a fourth gene (*Irx3*) that has been shown to function in this network (Fig. 3B) (Balaskas et al., 2012; Briscoe et al., 2000; Dessaud et al., 2007; Novitch et al., 2001). This allowed us to investigate two distinct gene expression boundaries (for *Nkx2.2* and *Olig2*) that have different responses to perturbations in their Gli binding affinity (Oosterveen et al., 2012; Peterson et al., 2012).

We use the same functional form for *ϕ* as previously described to determine the probability of polymerase occupancy (and therefore gene expression); however, to simplify the model we incorporate uniformly expressed TFs such as Sox2 into the expression for the basal level of polymerase binding for each gene. We assume that GliA acts a strong activator (c_AP_=10), that GliR acts as a strong repressor (c_RP_=0) and that the four TFs (Pax6, Olig2, Nkx2.2 and Irx3) are strong repressors, with zero cooperativity terms. For each gene repressed by one or more of these four TFs we include two binding sites. These act equally and independently, without cooperative binding, to repress polymerase binding. The inclusion of two independent binding sites provides the necessary non-linearity to generate sharp boundaries in gene expression patterns (Balaskas et al., 2012; Manu et al., 2009) and is consistent with the multiple binding motifs in experimental data (Oosterveen et al., 2012). We assume that the probability of gene expression, *ϕ*, is directly proportional to the rate of transcription.

The resulting ordinary differential equation (ODE) network model has the following form:9
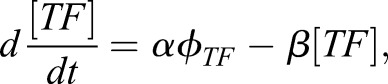
where [TF] represents the protein concentration of either Pax6, Olig2, Nkx2.2 or Irx3 and *φ_TF_* is the thermodynamic function describing polymerase occupancy for each of the respective genes. *α* is the production rate of the protein and *β* the degradation rate.

The thermodynamic functions are given by:
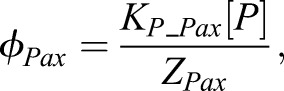

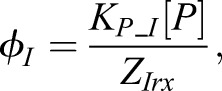

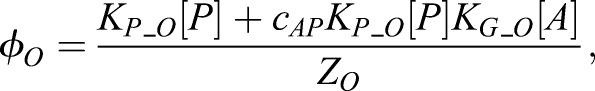

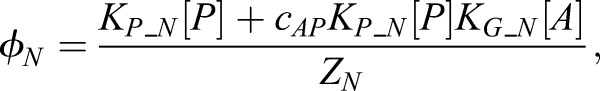

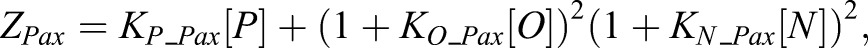

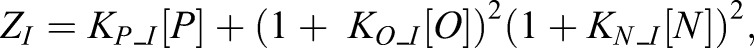
9
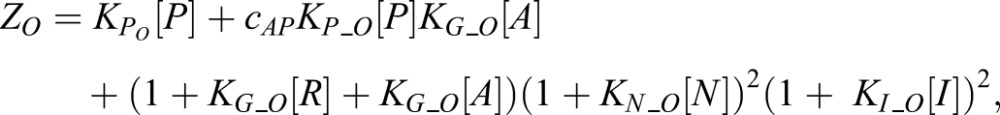

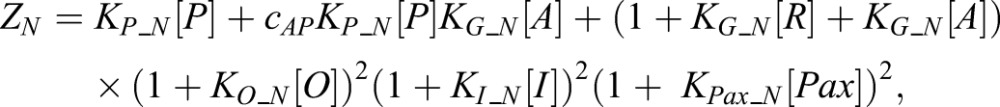
where [Pax], [O], [N], [I] represent the protein concentration of Pax6, Olig2, Nkx2.2 and Irx3 respectively. K_P_Pax_, K_P_O_, K_P_N_ and K_P_I_ represent the polymerase binding affinities for each of their enhancers. K_G_O_ and K_G_N_ represent the binding affinity of GliA and GliR for enhancers of *Olig2* and *Nkx2.2*. K_O_Pax_ denotes the binding affinity of Olig2 for the *Pax6* enhancer; other TF to TF binding affinities follow the same notation. We implemented the model within a GliA/R gradient (Fig. 2B).

As many of the parameters are unknown, we used established Bayesian methodology (Toni et al., 2009, Liepe et al., 2010; Liepe et al., 2014) to identify the distribution of parameters sets (posterior distributions) that could qualitatively reproduce the wild-type (WT) expression pattern observed in the ventral neural tube (Fig. 3A) (Alaynick et al., 2011; Balaskas et al., 2012), whereby Nkx2.2 is expressed most ventrally, followed by Olig2 and then Irx3, with dorsal Pax6 expression gradually decreasing across the Olig2 domain. We further constrained the parameter search by requiring the model to reproduce all of the observed null mutant phenotypes (Balaskas et al., 2012; Briscoe et al., 2001; Novitch et al., 2001) (Fig. 3D). This included the effect of simultaneous removal of both GliA and GliR [experimentally simulating *Shh^−/−^;Gli3^−/−^* or *Gli2^−/−^;Gli3^−/−^* compound mutants (Litingtung and Chiang, 2000; Persson et al., 2002)]. In these embryos, Nkx2.2 expression is absent but Olig2 expression is observed in ventral regions of the neural tube, indicating that basal (Gli-independent) activation of Olig2 is sufficient for its expression.

We defined a distance function that compares simulated patterns with idealised representations of the WT and mutant patterns described above. The Bayesian method (see Materials and Methods and Fig. 3C for an overview of the process) enabled us to obtain a representation of the parameter space for which the model can approximate the observed phenotypes (i.e. achieving an appropriately low distance score for each of the targets; supplementary material Fig. S4A). We fixed the production and degradation rates of the TFs (*α*=*β*=2) in order to investigate the effect of the other parameters in the model. For each of the unknown parameters, which represent the binding affinities of the components and the concentration of polymerase, we searched over a prior parameter space spanning five orders of magnitude, sampling on a log scale between −3 and 2.

A typical simulation with a parameter set drawn from the full posterior distribution is shown in Fig. 3D. Consistent with the *in vivo* observations (Balaskas et al., 2012; Dessaud et al., 2007), the genes in the WT case are expressed in the temporal sequence: *Pax6*+*Irx3→Olig2→Nkx2.2* in the future p3 (Nkx2.2-expressing) domain (Fig. 3E). The marginal posterior parameter distributions (Fig. 3F; supplementary material Fig. S4B shows the joint posterior distributions) revealed that different strengths of binding are required among the different TFs. For example, Nkx2.2 binds relatively strongly to *Olig2* and *Pax6*, whereas Olig2 binds weakly to *Nkx2.2* and *Pax6* (the marginal posterior distributions have mean values of K_N_O_=57.6, K_N_P_=39.6, K_O_N_=3.3, K_O_P_=1.9). This is consistent with previous observations suggesting that Nkx2.2 acts as a stronger repressor than Olig2 or Pax6 in order to avoid oscillations in gene expression levels (Balaskas et al., 2012; Panovska-Griffiths et al., 2013).

**Fig. 4.**
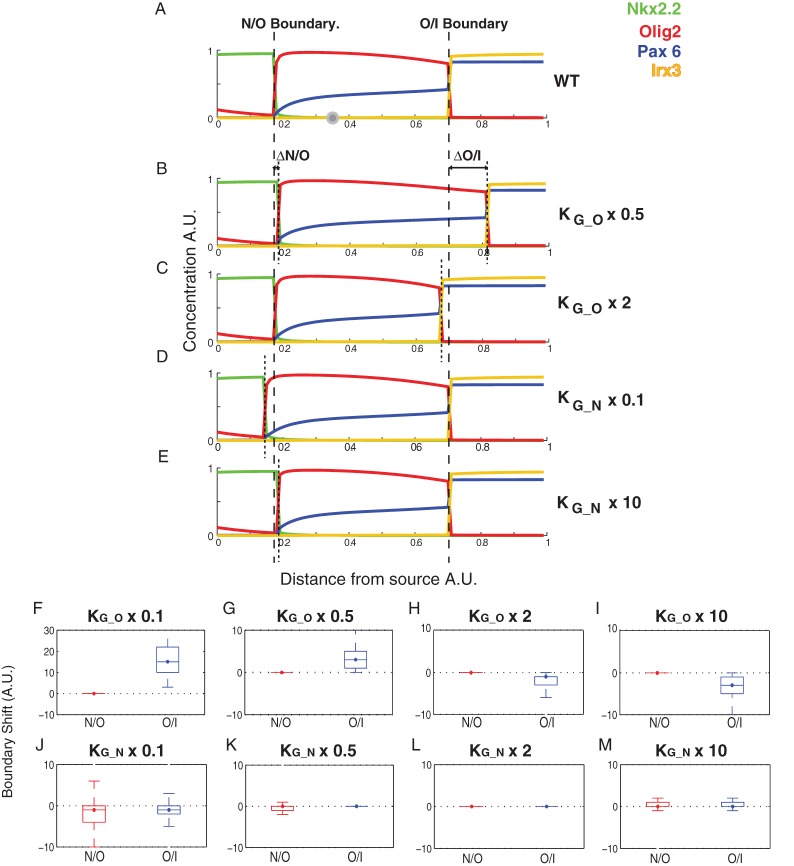
**Boundary shifts caused by perturbations to Gli binding in the network model.** (A) The steady-state WT pattern produced by the parameter set described in Fig. 3C after 100 h of Shh signalling. The neutral position, θ=0, for Olig2 and Nkx2.2 is indicated by the solid grey circle. The Nkx2.2/Olig2 (N/O) and Olig2/Irx3 (O/I) boundaries are indicated by dashed lines. (B-E) The patterns obtained when the model was simulated with perturbations to GBS affinity for either Olig2 or Nkx2.2. The resulting boundary shifts (ΔN/O and ΔO/I) are indicated by dotted lines. A twofold reduction (B) or increase (C) in the affinity of the GBS in the *Olig2* enhancer results in a shift in the ventral boundary away (B) or towards (C) the morphogen source, and leads to an expansion (B) or contraction (C) in the overall domain size. A tenfold reduction (D) or increase (E) in the affinity of the GBS in the *Nkx2.2* enhancer results in a slight reduction (D) or expansion (E) in its range of expression. (F-M) The positions of the Nkx2.2/Olig2 (N/O) and Olig2/Irx3 (O/I) boundaries were calculated using the algorithm described in the Materials and Methods. The shift in each boundary (ΔN/O and ΔO/I) caused by the perturbation to Gli binding affinity was calculated. The distribution of these boundary shifts from among the full posterior population of parameter sets is shown as a box blot (the median is identified by a large dot with strikethrough solid line, boxes are 25th and 75th percentiles, whiskers encompass ∼99% of the population, outliers are not shown). A population of 100,000 parameter sets was explored (derived by resampling the 1000 weighted particles obtained from ABC-SysBio). For each parameter set the model was perturbed from the WT case by altering the Gli binding affinity to either *Nkx2.2* or *Olig2*. The distribution of boundary shifts from the WT location is indicated for the N/O (red) and O/I (blue) boundaries. Gli binding to *Olig2* was reduced by a factor 0.1 (F) and 0.5 (G) or increased by a factor of 2 (H) and 10 (I). Gli binding to *Nkx2.2* was reduced by a factor 0.1 (J) and 0.5 (K) or increased by a factor of 2 (L) and 10 (M).

In order to establish the effect of changing different parameters, we analysed the change in position of the gene expression boundaries (Fig. 4; see Materials and Methods). For Olig2, decreasing GBS affinity significantly increased the range of its dorsal (Olig2/Irx3) expression boundary with Irx3, while simultaneously shifting the ventral boundary slightly more dorsally (Fig. 4B,F,G). Increasing GBS affinity significantly decreased the range of expression at the dorsal boundary (Fig. 4C,H,I). These shifts in Olig2 boundaries are consistent with the experimental observation that inhibiting the binding of both activator and repressor Gli proteins resulted in a dorsal shift of the Olig2 domain (Oosterveen et al., 2012). Notably, Olig2 expression, in the model, spanned the neutral position (*θ*=0 at *x*∼0.35, as denoted by the solid grey circle in Fig. 4A). Thus, its dorsal boundary was located in the region of the gradient where one would predict an increase in gene expression if GBS affinity were decreased (resulting in a dorsal expansion in the Olig2 domain due to the increased repression of Irx3 and Pax6). Moreover, the Olig2 ventral boundary was located in a region where decreasing GBS affinity would result in a decrease in gene expression (allowing the Nkx2.2 domain to expand dorsally due to the derepression at this boundary caused by the reduction in Olig2).

In the case of Nkx2.2, decreasing the affinity restricted Nkx2.2 expression more ventrally (Fig. 4D,J,K), whereas increasing the affinity expanded its expression dorsally (Fig. 4E,L,M). This is also consistent with the experimental data (Oosterveen et al., 2012; Peterson et al., 2012). Together, this analysis demonstrates that changes in GBS affinity of the genes in the network result in the same behaviour as predicted for a solitary gene.

This system also displays hysteresis (Balaskas et al., 2012), such that the steady state is maintained if the signal is reduced once the network has reached steady state (supplementary material Fig. S5). Both the Nkx2.2/Olig2 boundary and the Olig2/Irx3 boundary are robust to significant reductions (up to 80%) in the input signal of GliA once steady state is achieved (supplementary material Fig. S5A-D). By contrast, both boundary positions are highly sensitive to levels of GliA at the start of a simulation (supplementary material Fig. S5E-H).

We also asked how gene expression is affected if the strength of the uniform input is altered (supplementary material Fig. S6A-J). We considered two cases. First, changing the basal signal for all four genes simultaneously (by changing the level of [P]; supplementary material Fig. S6A,B) had relatively small effects on the pattern. This suggests robustness in the system that preserves the pattern when the concentration of a uniform input parameter is changed. By contrast, altering the basal input for individual genes (by changing either K_P_Pax_, K_P_Irx_, K_P_O_ or K_P_N_; supplementary material Fig. S6C-J) significantly altered the pattern of gene expression. For example, a twofold increase in the basal input to Olig2 dramatically expanded the size of its expression domain, whereas a twofold decrease resulted in a dramatic decrease in expression (supplementary material Fig. S6I,J). Thus, altering the number or strength of uniform inputs modifies the spatial pattern of gene expression.

Moreover, as demonstrated previously (Balaskas et al., 2012; Panovska-Griffiths et al., 2013), changing the binding affinities of the TFs forming the cross-repressive network altered the pattern and behaviour of gene expression (supplementary material Fig. S6K-BB). Together, these mechanisms could be exploited in the neural tube to modify the gene expression domains at different anterior-posterior positions, thereby providing a means to generate and fine-tune different patterns of gene expression without changing the morphogen gradient itself. A similar strategy could be exploited during evolution to alter the patterns of gene expression between species. This offers a level of flexibility to pattern formation that could have favoured the use of this type of morphogen system during the evolution of tissue patterning mechanisms.

## DISCUSSION

**Fig. 5.**
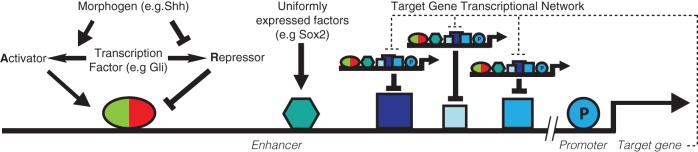
**Model for morphogen target gene regulation.** Shown is a morphogen-regulated gene in which an enhancer integrates the activity of three distinct classes of TFs. The activity of the MR-TF, which acts as a bifunctional activator-repressor, is determined by the level of morphogen signalling and provides positional information. Uniformly expressed TFs, exemplified by Sox2, are active throughout the tissue and contribute to the basal level of activity of a target gene. The target genes that are dynamically regulated downstream of the morphogen feedback into gene regulation. Each of these inputs can comprise multiple individual TFs with either inhibitor or activator function and the modelling framework is extendable to account for multiple inputs to a single gene.

Taken together, the model provides a straightforward way to capture the idea that the activity of target genes is determined by the combined input of the transcriptional effectors. This view of patterning is consistent with the notion that cis-regulatory elements integrate the activity of TFs to determine the probability and/or rate of transcription (Davidson, 2006). Three distinct classes of inputs can be defined: the MR-TF, the activity of which is determined by the distribution of the morphogen in the tissue; uniformly expressed TFs that are active throughout the tissue; and morphogen-controlled target genes that are dynamically regulated downstream of the morphogen. Each of these inputs can comprise multiple individual TFs with either inhibitor or activator function (Fig. 5). The thermodynamic regulation function provides a method for logically combining these different regulatory factors.

The analysis reveals how the cross-regulatory network is able to establish sharp gene expression boundaries (among target genes that, if expressed in isolation, would have a graded or uniform expression profile). By explicitly including the bifunctional forms of Gli we were able to account for the different boundary shifts that arise in different Shh targets when Gli binding affinity is perturbed. Notably, these differences could occur in genes containing a single GBS with the same affinity for the repressor and activator forms of Gli. In particular, the model predicts that the Olig2 expression domain must overlie the neutral point in the gradient in order to explain the different shifts observed in its ventral and dorsal boundaries. Moreover, by using the Bayesian methodology to explore the parameter space we were able to make specific predictions about the relative strength of binding affinities as well the effects of different parameter perturbations.

Previously, the thermodynamic modelling approach has been used to explain how uniformly expressed transcriptional activators can collaborate with latent MR-TFs that are activated by a morphogen (Kanodia et al., 2012). Here we show that the same framework can be extended to describe transcriptional networks regulated by a morphogen, thereby allowing the function and architecture of cis-regulated elements and the dynamics of a gene regulatory network to be explored simultaneously. Thus, this modelling formalism provides a unifying theoretical framework with which to analyse differential gene expression in developing tissues.

## MATERIALS AND METHODS

### Approximate Bayesian computation (ABC)

Simulations of the gene expression functions were carried out using Mathematica 9.0 (Wolfram Research). The network model was analysed using ABC-SysBio (Liepe et al., 2010; Liepe et al., 2014), an ABC (Toni et al., 2009) suite designed to run in parallel on graphics processing units (GPUs) using the python package cuda-sim (Zhou et al., 2011). The input gradient of GliA and GliR described in Fig. 2 was discretised into 100 positions. At each position the ODEs describing the TF dynamics were solved to steady state (100 hours) for each parameter set. Mutant patterns were generated by setting the levels of production of the relevant TF to zero or, for *Gli*^–/–^, by setting the binding affinity of Gli to zero. A distance function in the software compared the WT and mutant simulated patterns with a set of stereotyped targets (Fig. 3D). A point was accrued if the simulated protein concentration differed from the target by more than 0.2 A.U. at each of the 100 discrete positions in the gradient. The total mean distance score was obtained by summing the score for each gene at each position and dividing by the total number of positions scored. Hence, a simulated pattern that perfectly matched a target would score zero and one that completely mismatched the target would score 1. The posterior parameter distribution was estimated using 1000 particles (parameter sets). It uses a sequential Monte Carlo (SMC) algorithm to efficiently search the parameter space [for details, see Liepe et al. (2010)]. Initially, a pilot run was performed in which the target was the WT pattern. The priors were then updated with the posterior distributions obtained from this fit and then the WT and mutant patterns were used together as targets; this was used to speed up the search process (the total time to obtain the approximate posterior distributions was around 3 weeks running on a single GPU). After this time the mean distance scores illustrated in supplementary material Fig. S4A were obtained. The joint and marginal parameter probability distributions based on the weighted particles (supplementary material Fig. S4B) were plotted using ksdensity and meshgrid functions in Matlab (MathWorks).

To determine the effect of parameter perturbations, gene expression boundaries were compared with the WT pattern (Fig. 4; supplementary material Figs S5, S6). A resampled posterior population of 100,000 parameters sets was derived from the final 1000 weighted particles. For each pattern obtained from this set, the gene expression boundaries were defined using the following algorithm that searched for the maximum or minimum positions at which expression levels of the different TFs were higher or lower than each other; specifically, the Nkx2.2 (N)/Olig2 (O) boundary was defined as: mean[max(N>O in region N>I), min(O>N in region O>I)]. The Olig2 (O)/Irx3 (I) boundary was defined as: mean[max(O<I in region O>N), min(I>O in region I>N)]. If no positions in the pattern satisfied the requirements for both the N/O and O/I boundary, or if they were located in reverse order of the WT pattern, then that parameter set was deemed as non-patterning. For each ‘patterning’ solution the distance between the WT and perturbed boundary was determined. The boxplots in Fig. 4 and in supplementary material Figs S5 and S6 showing the distribution of these distances for each boundary were derived using the Matlab boxplot function. The boxes show the median and 25th (q1) and 75th (q3) percentiles, the whiskers extend to q3+1.5(q3–q1), covering approximately 99% of the normally distributed data (outliers are not shown).

## Supplementary Material

Supplementary Material
