# Supplementary material for: A theoretical framework for the regulation of Shh morphogen-controlled gene expression
Source: Development. 2014 Oct;141(20):3868–78. doi: 10.1242/dev.112573 (PMC4197706; doi:10.1242/dev.112573)
Supplement: Supplementary Material [file supp_141_20_3868__index.html]

A theoretical framework for the regulation of Shh morphogen-controlled gene expression — Supplementary Material 

# A theoretical framework for the regulation of Shh morphogen-controlled gene expression

## DEV112573 Supplementary Material

**Files in this Data Supplement:**

- **Supplementary Material**
