# Supplementary material for: A theoretical framework for the regulation of Shh morphogen-controlled gene expression
Source: Development. 2014 Oct;141(20):3868–78. doi: 10.1242/dev.112573 (PMC4197706; doi:10.1242/dev.112573)
Supplement: Supplementary Material [file supp_141.20.3868_DEV112573.pdf]

Supplementary Methods

Section 1 – Comparing changes in GBS affinity with changes in the number of binding sites

In the main text we have focused on the relationship between binding site affinity and gene expression. Figure S1A summarizes how, in the single binding site model, changing the binding affinity, K (in this case over a two-orders of magnitude range) changes gene expression probability.

Altering the number of binding sites can also affect gene expression probability in this model. For the simplest case we assume that each GBS acts independently, that is there is no cooperative binding among the isoforms of Gli and for every bound GBS site there will be an additive reduction in the binding energy of polymerase (hence there will be a multiplicative effect on the gene expression probability function). To simplify the equations, we further assume that GliR acts a strong repressor (C_{RP}=0). Hence, for example, the expression for three GBS sites in this case will be:

(Equation S1)

$$\phi = \frac{K_P[P] + 3c_{AP}K_P[P]K[A] + 3K_P[P](c_{AP}K[A])^2 + K_P[P](c_{AP}K[A])^3}{Z}$$

where

$$Z = K_P[P] + 3c_{AP}K_P[P]K[A] + 3K_P[P](c_{AP}K[A])^2 + K_P[P](c_{AP}K[A])^3 + 1 + 3K[R] + 3(K[R])^2 + (K[R])^3 + 3K[A] + 3(K[A])^2 + (K[A])^3 + 6(K^2R.A) + 3(K^3R^2A) + 3(K^3A^2R)\ .$$

Figure S1B shows the effect of changing binding site number. This model displays a similar behaviour to changing the affinity of a single binding site. In both cases (Figure S1A-B) there is a neutral point at which there is no effect on the gene expression probability (which is at the basal level). At positions closer to the source of morphogen than the neutral point increasing the number of binding sites increases the probability of expression, whereas at positions

beyond the neutral point increasing binding site number decreases expression. The two models are therefore qualitatively similar. Indeed, changing binding affinity in the three-site model also has a similar effect to the single binding site model (Figure S1C).

Increasing the number of sites also results in an increasingly non-linear response to the graded input. It should be noted that the model described here represents an idealized case in which each bound GBS has the same cumulative effect on the total activation or inhibition – regardless of how many sites are already bound. It is potentially more likely that there would be some kind of diminishing effect so that each additional GBS that is bound has less effect on polymerase than those already bound. In this case adding more binding sites would make the gene response relatively less non-linear [data not shown].

Section 2 – A model in which transcription factor binding rates are unaffected by bound polymerase has a neutral point that is dependent on basal transcription levels.

The thermodynamic model, defined by equation 1 in the main text, explicitly describes a situation in which activators (or repressors) act by reducing (or increasing) the total energy of the state in which they are bound at the DNA together with polymerase – such that the total energy is less (or more) than the sum of the energies of the individually bound species. Thus, the presence of a bound activator increases the probability that free polymerase will bind. The model also implies that bound polymerase will act to increase the probability that free activator will bind (or reduce the probability of free repressor binding). This makes intuitive sense if one regards the DNA, transcription factor and promoter as single bound complex; however, it is unclear whether or not this is the most appropriate description of the underlying molecular processes in a cis-regulatory system. An alternative model would be to assume that an activator (or repressor) can increase (or reduce) the rate of polymerase binding - or alternatively reduce (or increase) unbinding - whilst the binding rate of the activator (or repressor) is unaffected by the presence of polymerase. In this case it is not straightforward to model the system using the thermodynamic framework because it is not possible to define a unique energy level associated with each bound state.

Instead, the probability of gene expression ϕ, can be expressed such that:

$$\begin{aligned}\phi &= \mathbb{P}(P_{bound}) \\ &= \mathbb{P}(P_{bound}|GBS_{unbound})\mathbb{P}(GBS_{unbound}) + \mathbb{P}(P_{bound}|A_{bound})\mathbb{P}(A_{bound}) + \mathbb{P}(P_{bound}|R_{bound})\mathbb{P}(R_{bound})\end{aligned}$$

Since in this model the binding of GliA and GliR are independent of P, a simple consideration of the binding reactions gives:

$$\mathbb{P}(GBS_{unbound}) = \frac{1}{1 + K_A[A] + K_R[R]}$$

$$\mathbb{P}(A_{bound}) = \frac{K_A[A]}{1 + K_A[A] + K_R[R]}$$

$$\mathbb{P}(R_{bound}) = \frac{K_R[R]}{1 + K_A[A] + K_R[R]}$$

We assume that A and R will change the binding constant of P by the factors c_{AP} and c_{AR} respectively. Therefore we obtain:

$$\mathbb{P}(P_{bound}|GBS_{unbound}) = \phi_{basal} = \frac{K_P[P]}{1 + K_P[P]}$$

$$\mathbb{P}(P_{bound}|A_{bound}) = \frac{c_{AP}K_P[P]}{1 + c_{AP}K_P[P]}$$

$$\mathbb{P}(P_{bound}|R_{bound}) = \frac{c_{RP}K_P[P]}{1 + c_{RP}K_P[P]}$$

This gives the expression for ϕ

(Equation S2)

$$\phi = \frac{K_P[P]}{1 + K_A[A] + K_R[R]}\left[\frac{1}{1 + K_P[P]} + \frac{c_{AP}K_A[A]}{1 + c_{AP}K_P[P]} + \frac{c_{RP}K_R[R]}{1 + c_{RP}K_P[P]}\right]$$

We can apply a similar logic as was applied previously to determine how changes to the Gli binding affinity, $(K \equiv K_A = K_R)$ will affect ϕ. We derive:

(Equation S3)

$$\frac{d\phi}{dK} = \frac{K_P[P]\,(\,[A](c_{AP} - 1)(1 + c_{RP}K_P[P]) + [R](c_{RP} - 1)(1 + c_{AP}K_P[P])\,)}{(1 + K_P[P])(1 + c_{AP}K_P[P])(1 + c_{RP}K_P[P])(1 + K[A] + K[R])^2}$$

In this case the neutral point, where $\frac{d\phi}{dK} = 0$, occurs at $\theta' = 0$, where

(Equation S4)

$$\theta' \equiv [A](c_{AP} - 1)(1 + c_{RP}K_P[P]) + [R](c_{RP} - 1)(1 + c_{AP}K_P[P]) \ .$$

At positions where $\theta' > 0$ increasing K will increase ϕ. Conversely at position where $\theta' < 0$ increasing K will decrease ϕ. In this model the neutral point ($\theta' = 0$) is not only determined by the gradient of GliA and GliR and their relative strengths of activation and repression, but also depends on the levels of polymerase in the system. The neutral point still defines the position in the gradient at which changing the levels of binding of Gli has no effect on their combined activating or repressing power – and indeed, at this position gene expression is still at basal levels. However, in contrast to the thermodynamic model described in the main text (equation 1) the actual position of the neutral point is no longer independent of the basal level of gene expression. We can see the relationship between the model parameters and the neutral point in Figure S2, where numerical examples are shown for different parameter sets.

Although this model exhibits these subtle differences to the thermodynamic model described in the main text, the fundamental result still holds; close to the source, increasing Gli binding affinity to the enhancer will increase gene expression probability and further from the source it will decrease. Determining precisely which molecular model of gene expression is more appropriate will require further experimental investigation.

Section 3 – Gene expression probability can be expressed as a linear function of signal strength

It is possible to rearrange the thermodynamic model for gene expression probability in the presence of S (i.e Sox2) binding (Equation 7) so that it becomes a linear function of the graded signal of GliA and GliR.

This takes the form:

(Equation S5)

$$\varphi = \varphi_{basal} + P_b[\alpha(\varphi_A - \varphi_R) - (\varphi_{basal} - \varphi_R)]$$

where,

$$\varphi = \frac{\phi}{1-\phi}$$

$$P_b = \frac{K_A[A] + K_R[R]}{1 + K_A[A] + K_R[R]}$$

$$\alpha = \frac{K_A[A]}{K_A[A] + K_R[R]}$$

$$\varphi_{basal} = \frac{1 + c_{SP}K_S[S]}{1 + K_S[S]} K_P[P]$$

$$\varphi_A = \frac{1 + c_{SP}K_S[S]}{1 + K_S[S]} c_{AP}K_P[P]$$

$$\varphi_R = \frac{1 + c_{SP}K_S[S]}{1 + K_S[S]} c_{AR}K_P[P]$$

We can regard φ as the ratio of the probability of polymerase being bound (and hence the probability of gene expression) and the probability of it being unbound. P_b is a measure of how much Gli will be bound in the absence of polymerase. If K_A=K_R and total Gli =constant=[A]+[R] then P_b will be a spatially

uniform constant. In this case α is representative of the gradient and defines the proportion of activating Gli to the total Gli. The remaining terms, $\varphi_{basal}, \varphi_A, \varphi_R$ contain only parameters that are spatially uniform and independent of Gli. Thus, φ will be linear in α.

In the case described in Supplemental section 2, where Gli binding is independent of Polymerase binding, we can also incorporate the binding of S to obtain the expression:

(Equation S6)

$$\phi = \phi_{basal} + P_b[\alpha(\phi_A - \phi_R) - (\phi_{basal} - \phi_R)]$$

where,

$$\phi = probability\ of\ gene\ expression$$

$$\phi_{basal} = \left[\frac{1}{1 + K_P[P]} + \frac{c_{SP}K_S[S]}{1 + c_{SP}K_P[P]}\right] K_P[P]$$

$$\phi_A = \left[\frac{1}{1 + c_{AP}K_P[P]} + \frac{c_{SP}K_S[S]}{1 + c_{AP}c_{SP}K_P[P]}\right] c_{AP}K_P[P]$$

$$\phi_R = \left[\frac{1}{1 + c_{RP}K_P[P]} + \frac{c_{SP}K_S[S]}{1 + c_{RP}c_{SP}K_P[P]}\right] c_{RP}K_P[P]$$

P_b and α are the same as in previous case. In this model, where Gli binding is independent of polymerase occupancy (in contrast to the thermodynamic model above) ϕ will be linear in α (when P_b is a constant). This therefore highlights a way in which these models could be distinguished experimentally.

Section 4 - More complex models of Gli binding can change the position of the neutral point

Other mechanisms have been proposed to explain the negative shift in boundary position caused by increasing the binding site affinity in a morphogen target gene (Parker et al., 2011; White et al., 2012). Notably in the wing disc, perturbations in the Dpp enhancer, a target of Hh, indicated that when Ci binding affinity was increased there was a shift in the position at which gene expression exceeded the basal (Ci independent) level of expression. This is in subtle contrast to the model in Equation 1 (or Equation S1) where the point at which gene expression equals basal levels, also defines the neutral point at which gene expression is independent of GBS affinity. Here we present those adaptations to the model that have been shown to reproduce this disruption to the neutral point behaviour.

Differential binding affinity

The first simple modification to the single binding site model described in Equation 1 is that the activator and repressor forms of Gli (or Ci in this case) could bind with different affinities to different enhancer regions. In particular for a shift towards the source in the position at which gene expression exceeds basal levels, this requires:

$$\frac{K_{R\_high}}{K_{R\_low}} > \frac{K_{A\_high}}{K_{A\_low}},$$

where K_{R_low} and K_{A_low} are the binding affinities of the 'low' affinity enhancer to R and A respectively, and K_{R_high} and K_{A_high} the 'high' affinity enhancer. In this case it is intuitive to see that increasing binding affinity proportionally more for the repressor could lead to more overall repression relative to the basal level of expression. This is shown in Figure S3A where Equation 1 has been simulated (solid blue line) but in this case the activator affinity has been increased 2 fold and the repressor affinity 3 fold (dashed blue line).

In regions very close to the source the gene expression has still increased (as in Figure 2C) and similarly very far from the source gene expression has decreased with the increase in GBS affinities. However in contrast to Figure 2C the point at which gene expression is at basal levels, has also shifted towards the source.

Cooperative repressor binding

A second alternative model, favored by Parker et. al. (Driever et al., 1989; Parker et al., 2011; White et al., 2012) to explain the Dpp response, was one in which the binding affinities for A and R are the same but there is cooperative binding between the repressive form of Gli at multiple binding sites. We illustrate this model schematically in Figure S3B for a simplified case in which there are two binding sites (note that three Ci binding sites were identified in the Dpp enhancer and included in the original analysis of the wing disc data).

The function for ϕ in this case has the form:

(Equation S7)

$$\phi = \frac{K_P[P] + 2c_{AP}K_P[P]K_A[A] + 2c_{RP}K_P[P]K_R[R] + 2c_{AP}c_{RP}c_{AR}K_P[P]K_A[A]K_R[R] + c_{RR}K_P[P](c_{RP}K_R[R])^2 + c_{AA}K_P[P](c_{AP}K_A[A])^2}{Z}$$

where

$$Z = 1 + 2K_R[R] + 2K_A[A] + c_{RR}(K_R[R])^2 + c_{AA}(K_A[A])^2 + 2c_{AR}K_A[A]K_R[R] + K_P[P] + 2c_{AP}K_P[P]K_A[A] + 2c_{RP}K_P[P]K_R[R] + 2c_{AP}c_{RP}c_{AR}K_P[P]K_A[A]K_R[R] + c_{RR}K_P[P](c_{RP}K_R[R])^2 + c_{AA}K_P[P](c_{AP}K_A[A])^2$$

c_{RR}, c_{AA}, c_{AR}, represent the cooperativity factors for binding of RR, AA, RA at adjacent GBSs.

The basal level of expression is still the same as in Equation 2. Crucially in this model it can be shown analytically that if $c_{RR} \neq c_{AA}$ then the position at which the function ϕ is equal to the basal level of gene expression will be dependent on the GBS affinity K (in contrast to single binding site model).

A numerical example is illustrated in Figure S3C where $c_{RR}>>c_{AA}$. In this model there is an increase in gene expression close to the source and a decrease far from the source. However, now the position of gene expression above the basal (thin grey line) is shifted towards the gradient source when GBS affinity is increased. The cooperative binding of the inhibitor means that increasing GBS affinity increases the inhibition proportionally more than the activation.

The cooperative model was favored over the differential affinity model by (Parker et al., 2011) as it fitted their data well and also suggested an explanation for why genes with higher binding affinities may be expressed closer to the ligand source. However, as we have demonstrated this could simply be a result of differences in other signal levels acting on different genes. Distinguishing these models may be more difficult given the resolution of real data.

FIGURE LEGENDS

Figure S1 – The effects of changing GBS affinity and GBS site number.

A – The effect of varying GBS affinity in a single site model. Simulations of the model defined by equation 1 using the graded input described by Figure 2B, for different values of K (GBS affinity) as indicated. Remaining parameters are: [P]=1, K_P=1, c_{AP}=10, c_{RP}=0. The basal level of gene expression is shown by the solid grey line – this was set to a relatively high value of 0.5 to show the full range of response.

B – The effect of varying GBS site number. Simulations in which the number of GBS sites in the target gene is changed (as indicated by the number n in the figure). In each case it is assumed that each extra site occupied by activator produces an additive reduction in the polymerase binding energy. GliR acts a strong repressor (c_{RP}=0) so its binding will always unbind polymerase. Remaining parameters are: K=1, [P]=1, K_P=1, c_{AP}=10, c_{RP}=0. Increasing the number of binding sites has a similar qualitative effect to increasing the affinity of a single binding site (A).

C – The effect of varying binding site affinity, K, in a model with 3 GBSs. The model described by equation S1 simulated with K=1 and K=5 as indicated in the figure. Remaining parameters are: [P]=1, K_P=1, c_{AP}=10, c_{RP}=0.

Figure S2 – A model in which Gli binding rates are independent of polymerase occupancy has a distinct neutral point.

A – The same gradient defined in Figure 2B for activator [A] and repressor [R]. These are defined such that $A=e^{-x/1.5}$, R=1-A.

B-F – Simulations of the model defined by equation S2 for different sets of parameters as indicated. In contrast to Figure 2, the neutral point, at which gene expression probability is unchanged by changing K (highlighted by a yellow dot) is dependent on basal gene expression as well as the cooperativity terms c_{AP} and c_{RP}. Remaining parameters are: K_P=1. The basal level of gene expression is shown

by the solid grey line. The position at which the probability of A and R binding at the enhancer is equated is defined solely by the concentration gradient; this is indicated by the dashed vertical line, which does not necessarily coincide with the neutral point.

Figure S3 – Changing Gli binding affinities in models with either differential affinity or cooperative binding results in a shift in the position at which gene expression is at basal levels.

A – An implementation of Equation 1 with differential binding affinities for activator and repressor. In the solid line $K_{A\_low}=K_{R\_low}=1$. In the dashed line $K_{A\_high}=2$, $K_{R\_high}=3$. There is shift in the position at which gene expression is equal to the basal levels highlighted by the red and blue circles. The simulation is based on the spatial distribution of activator and repressor described in Figure 2B. Parameters are [P]=1, $K_P=1$, $c_{AP}=10$, $c_{RP}=0.1$.

B – A schematic showing all possible DNA binding configurations for the model described by Equation S7 where there are two GBSs at the enhancer. All transcriptionally active configurations are shown with black arrows. Inactive configurations are shown with crossed grey arrows.

C – An implementation of Equation S7 with cooperative binding between repressors also leads to a shift in the position at which gene expression is equal to the basal level. Parameters are [P]=1, $K_P=1$, $c_{AP}=10$, $c_{RP}=0.1$, $c_{AA}=1$, $c_{AR}=1$, $c_{RR}=10$. In the solid line K=1. In the dashed line K=5.

Figure S4 – The scores and posterior distribution obtained in the Bayesian analysis

A – The mean distance scores for the population of parameter sets used in this study. The scores represent the proportion of the 100 discrete positions in the gradient for which the simulated pattern of protein expression was a distance greater than 0.2A.U. from the target pattern. The scores have been broken down for each gene in each of the WT and mutant patterns (the black bars show the average score for all 4 genes). Generally the WT patterns fit the best – in all

cases a pattern of 3 distinct stripes of Nkx2.2, Olig2, Irx3 were achieved. The Pax6-/- and Irx3-/- were the least well fit. (See Figure 3D for an example of the simulated output).

B – The posterior parameter distribution obtained from ABC-SysBio. This approximately defines the multi dimensional parameter space for which the model is able to reproduce the target patterns (to within the specified distance score). The marginal distributions reflect the constraint on each individual parameter – representing a single dimension of parameter space. Histograms for each of the marginal distributions are plotted in black along the main diagonal. These are plotted on a log-scale x-axis ranging from -3 to 2 (the range of the initial prior search space). The height of the bars represents the relative density of the posterior parameter space occupied by each parameter. The joint distributions map the relative density of a two-dimensional slice of parameter space orthogonal to the two parameters in the row and column as indicated - both axes in this case range from -3 to 2 (the x-axis refers to the parameter in that column, the y-axis to the parameter in that row), density ranges from blue (low) to red (high). Note that in this case, no two parameters are tightly correlated and the joint distributions simply reflect the overlapping marginal distributions.

Figure S5 – Hysteresis in the network model.

A-H The box plots indicate the distribution of boundary shifts (as illustrated in Figure 4) from the WT condition under different perturbations to the level of GliA in the input signal. The total graded signal was maintained such that GliA+GliR=1 for all positions. The input gradient was altered by reducing the amount of GliA according the scaling factor indicated. This was either done from 0hours (E-H) or from 100hours (A-D) by which time the system had achieved a steady pattern. As expected, when GliA was reduced at the start of the simulation the boundaries shifted significantly (E-H). For perturbations to GliA after 100hours there was no significant change in the boundary positions for up to a 50% decrease in GliA (A-C) and a small change to some of the population after an 80% decrease (D). This provides evidence of hysteresis.

Figure S6 – Effect of perturbations to network model parameters.

A-BB The box plots indicate the distribution of boundary shifts (as illustrated in Figure 4) from the WT condition under the different perturbations A–B The basal input of polymerase [P] to all three genes was varied by either a factor 2 (A) or 0.5 (B). C-J The steady state patterns after the binding of polymerase to one of the three genes is varied by a factor of 2 or 0.5 as indicated in the Figures: (C, D) changing the affinity of polymerase for Nkx2.2; (E,F) changing the affinity of polymerase for Pax6; (G,H) changing the affinity of polymerase for Irx3; (I,J) changing the affinity of Polymerase for Olig2. K-BB The binding affinities among the different TFs were altered by factor of 2 or 0.5: (K,L) changing the affinity of Pax6 for the Nkx2.2 enhancer; (M, N) changing the affinity of Irx3 for the Olig2 enhancer; (O, P) changing the affinity of Nkx2.2 for the Olig2 enhancer; (Q, R) changing the affinity of Nkx2.2 for the Pax6 enhancer; (S, T) changing the affinity of Olig2 for the Pax6 enhancer; (U, V) changing the affinity of Irx3 for the Nkx2.2 enhancer; (W, X) changing the affinity of Nkx2.2 for the Irx3 enhancer; (Y, Z) changing the affinity of Olig2 for the Nkx2.2 enhancer; (AA, BB) changing the affinity of Olig2 for the Irx3 enhancer.

Figure S1

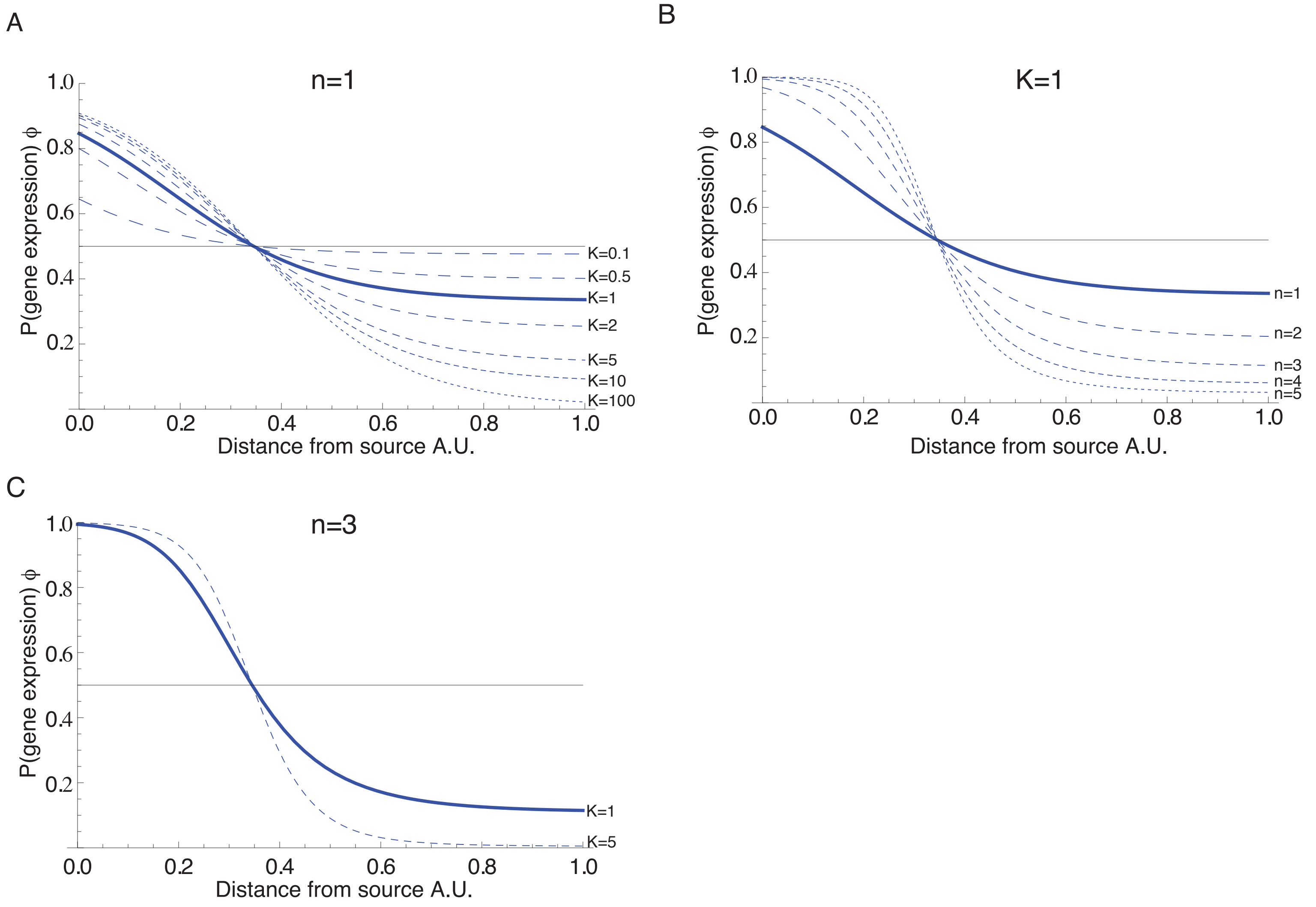
A
n=1
P(gene expression) ϕ
1.0
0.8
0.6
0.4
0.2
K=0.1
K=0.5
K=1
K=2
K=5
K=10
K=100
0.0
0.2
0.4
0.6
0.8
1.0
Distance from source A.U.
B
K=1
P(gene expression) ϕ
n=1
n=2
n=3
n=4
n=5
Distance from source A.U.
C
n=3
P(gene expression) ϕ
K=1
K=5
Distance from source A.U.

Figure S2

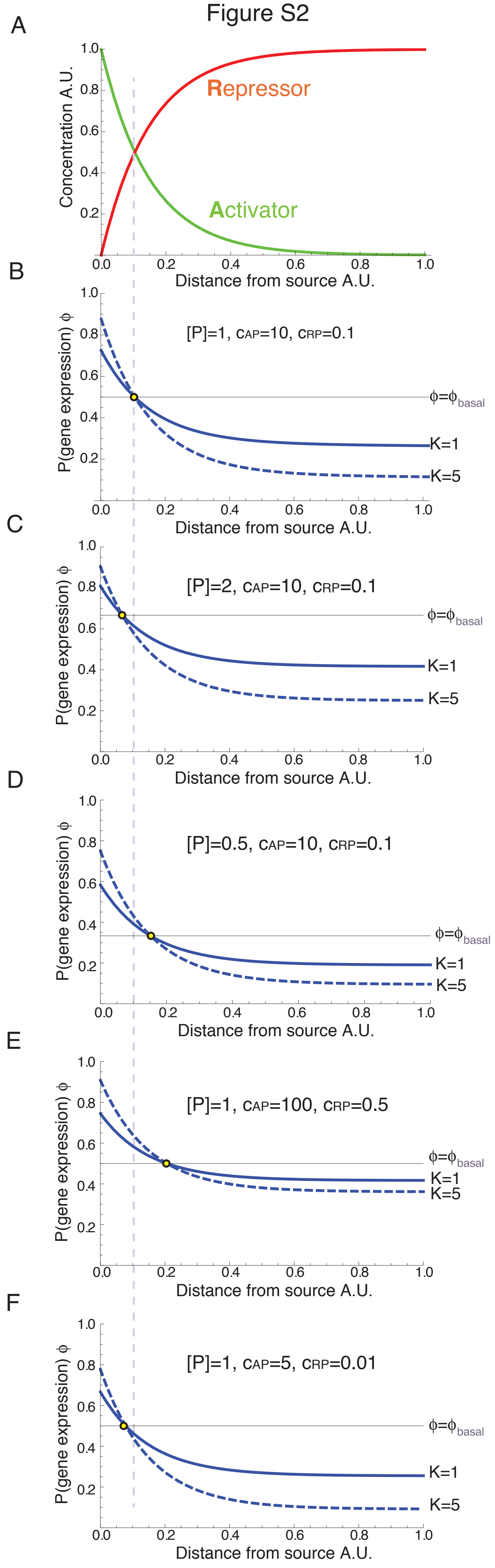

A
Concentration A.U.
Repressor
Activator
Distance from source A.U.
B
P(gene expression) ϕ
[P]=1, cAP=10, cRP=0.1
ϕ=ϕbasal
K=1
K=5
Distance from source A.U.
C
P(gene expression) ϕ
[P]=2, cAP=10, cRP=0.1
ϕ=ϕbasal
K=1
K=5
Distance from source A.U.
D
P(gene expression) ϕ
[P]=0.5, cAP=10, cRP=0.1
ϕ=ϕbasal
K=1
K=5
Distance from source A.U.
E
P(gene expression) ϕ
[P]=1, cAP=100, cRP=0.5
ϕ=ϕbasal
K=1
K=5
Distance from source A.U.
F
P(gene expression) ϕ
[P]=1, cAP=5, cRP=0.01
ϕ=ϕbasal
K=1
K=5
Distance from source A.U.

Figure S3

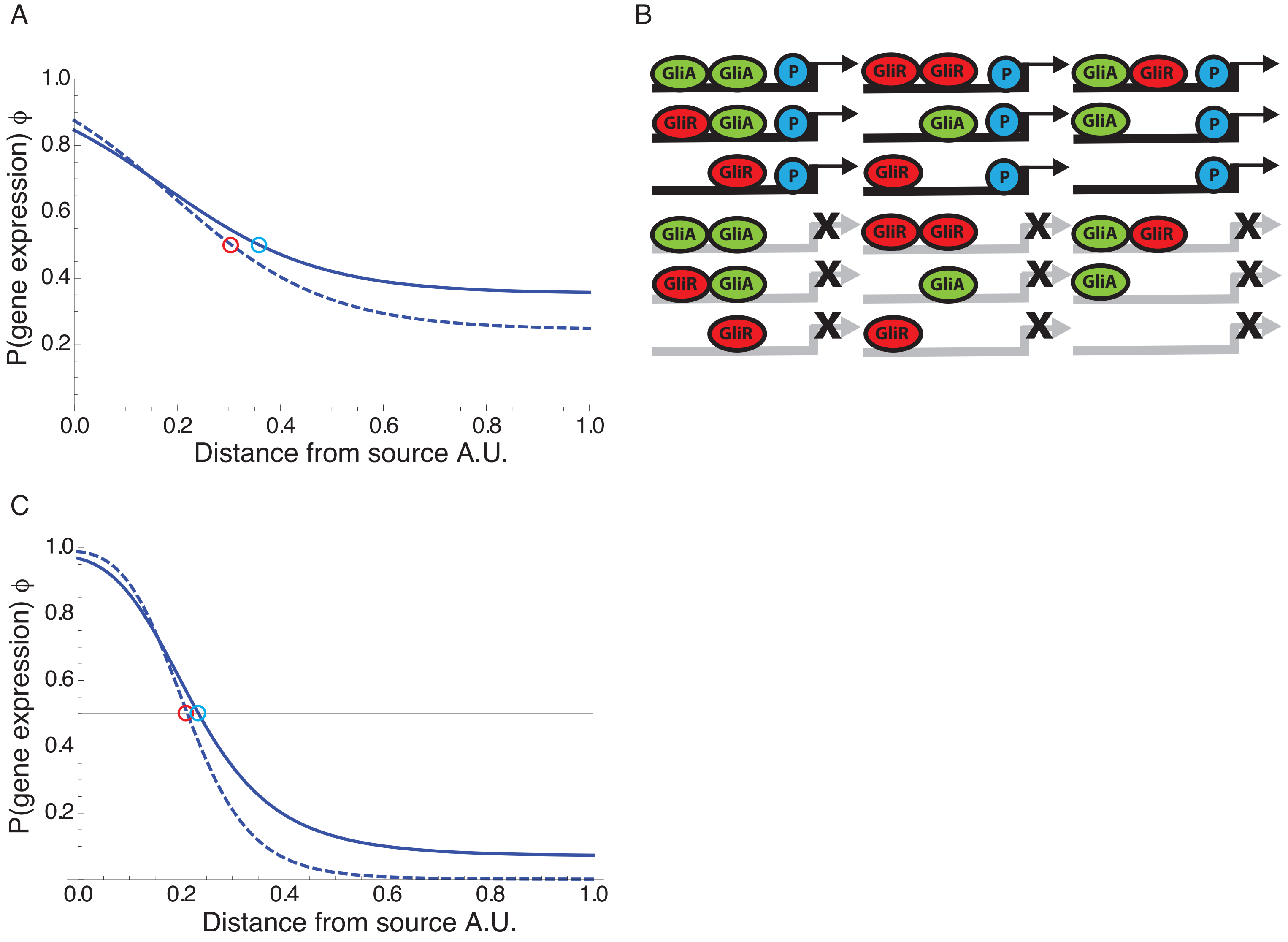
A
1.0
0.8
0.6
0.4
0.2
P(gene expression) ϕ
0.0
0.2
0.4
0.6
0.8
1.0
Distance from source A.U.
B
GliA
GliR
P
C
1.0
0.8
0.6
0.4
0.2
P(gene expression) ϕ
0.0
0.2
0.4
0.6
0.8
1.0
Distance from source A.U.

Figure S4

A

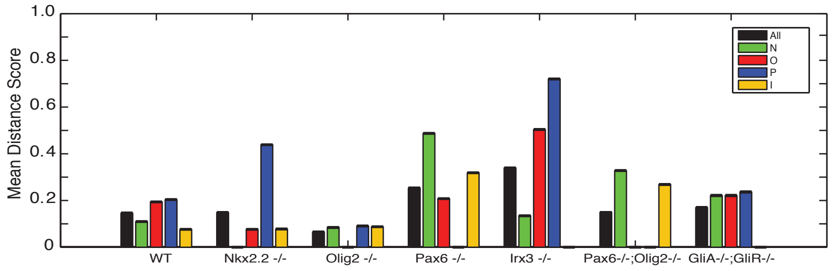
1.0
0.8
0.6
0.4
0.2
0
Mean Distance Score
All
N
O
P
I
WT
Nkx2.2 -/-
Olig2 -/-
Pax6 -/-
Irx3 -/-
Pax6-/-;Olig2-/-
GliA-/-;GliR-/-

B

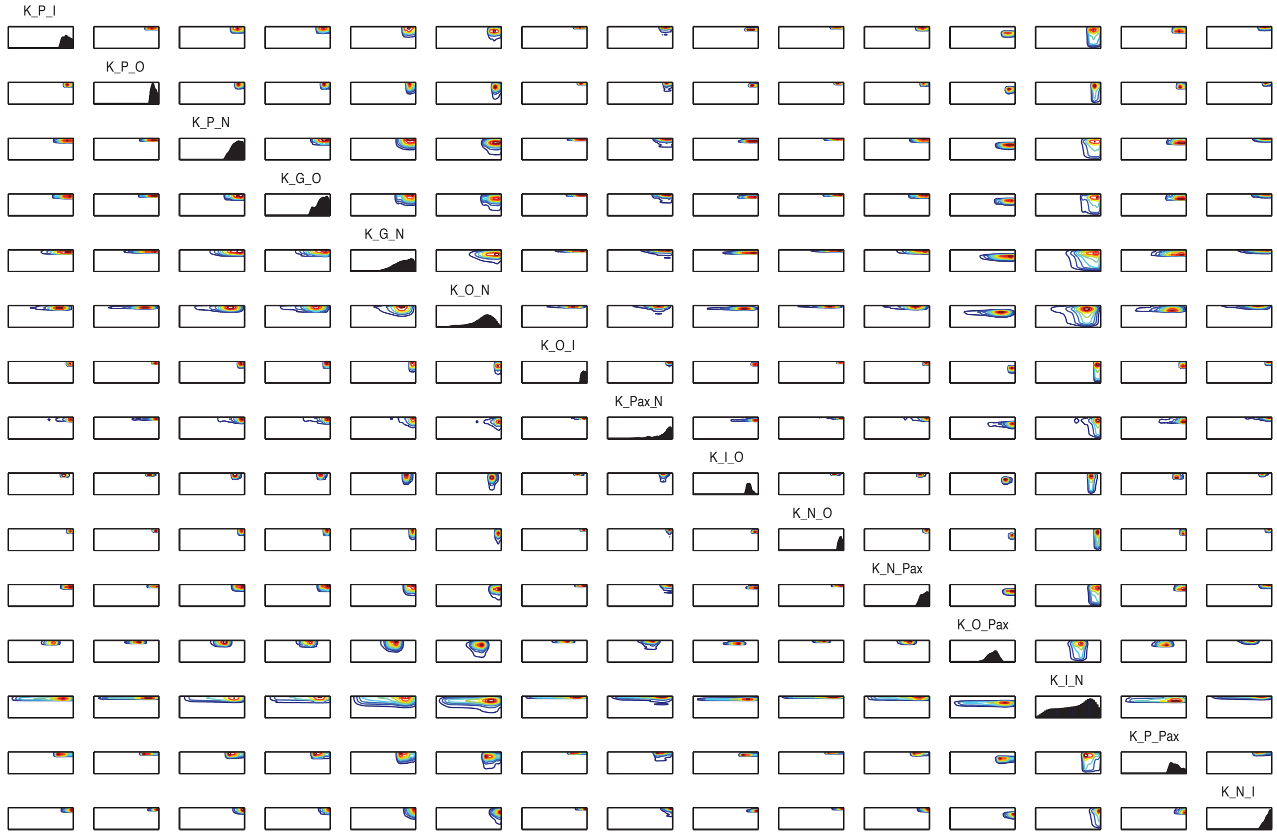
K_P_I
K_P_O
K_P_N
K_G_O
K_G_N
K_O_N
K_O_I
K_Pax_N
K_I_O
K_N_O
K_N_Pax
K_O_Pax
K_I_N
K_P_Pax
K_N_I

Figure S5

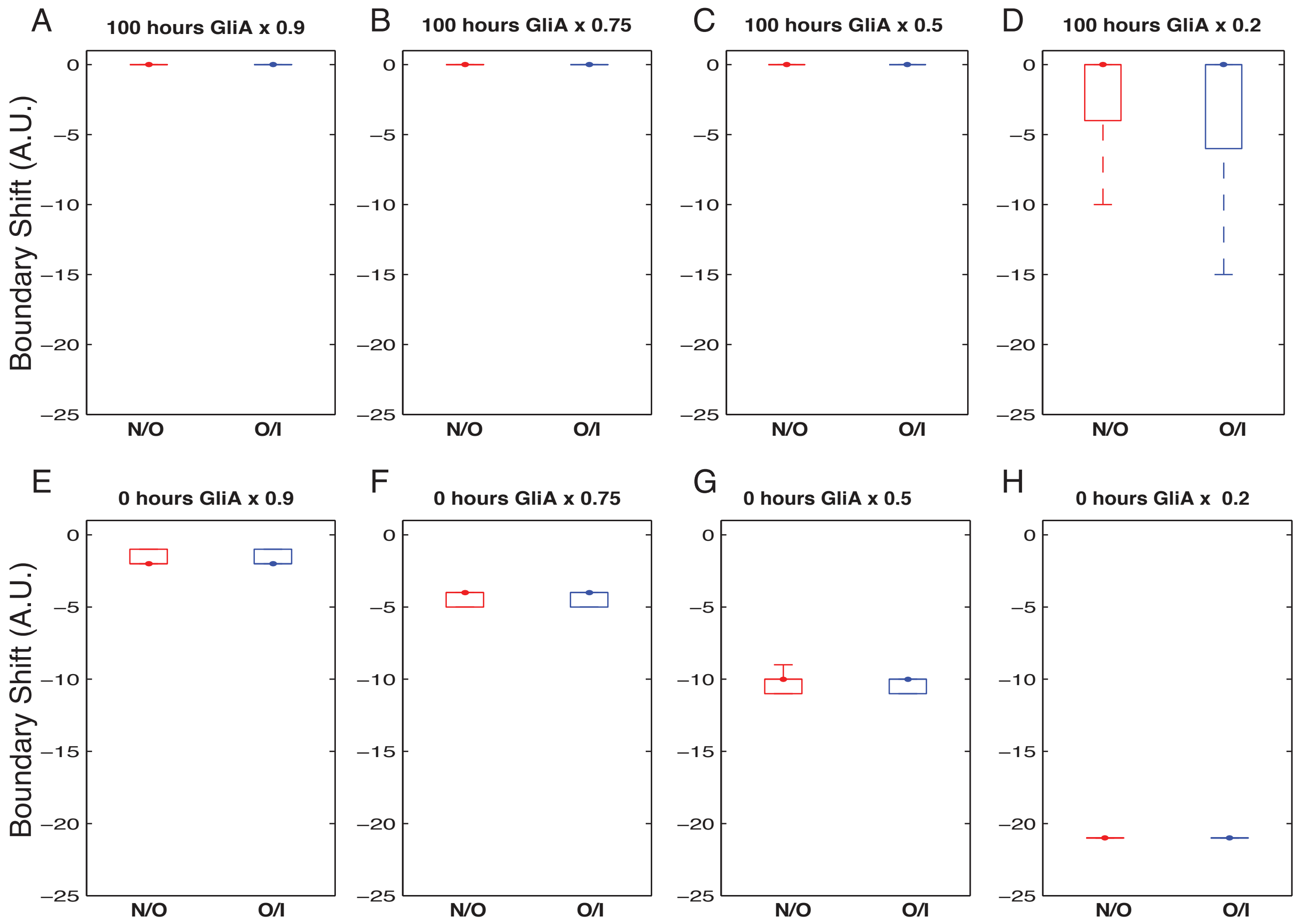
A
100 hours GliA x 0.9
B
100 hours GliA x 0.75
C
100 hours GliA x 0.5
D
100 hours GliA x 0.2
E
0 hours GliA x 0.9
F
0 hours GliA x 0.75
G
0 hours GliA x 0.5
H
0 hours GliA x 0.2
Boundary Shift (A.U.)
0
−5
−10
−15
−20
−25
N/O
O/I

Figure S6

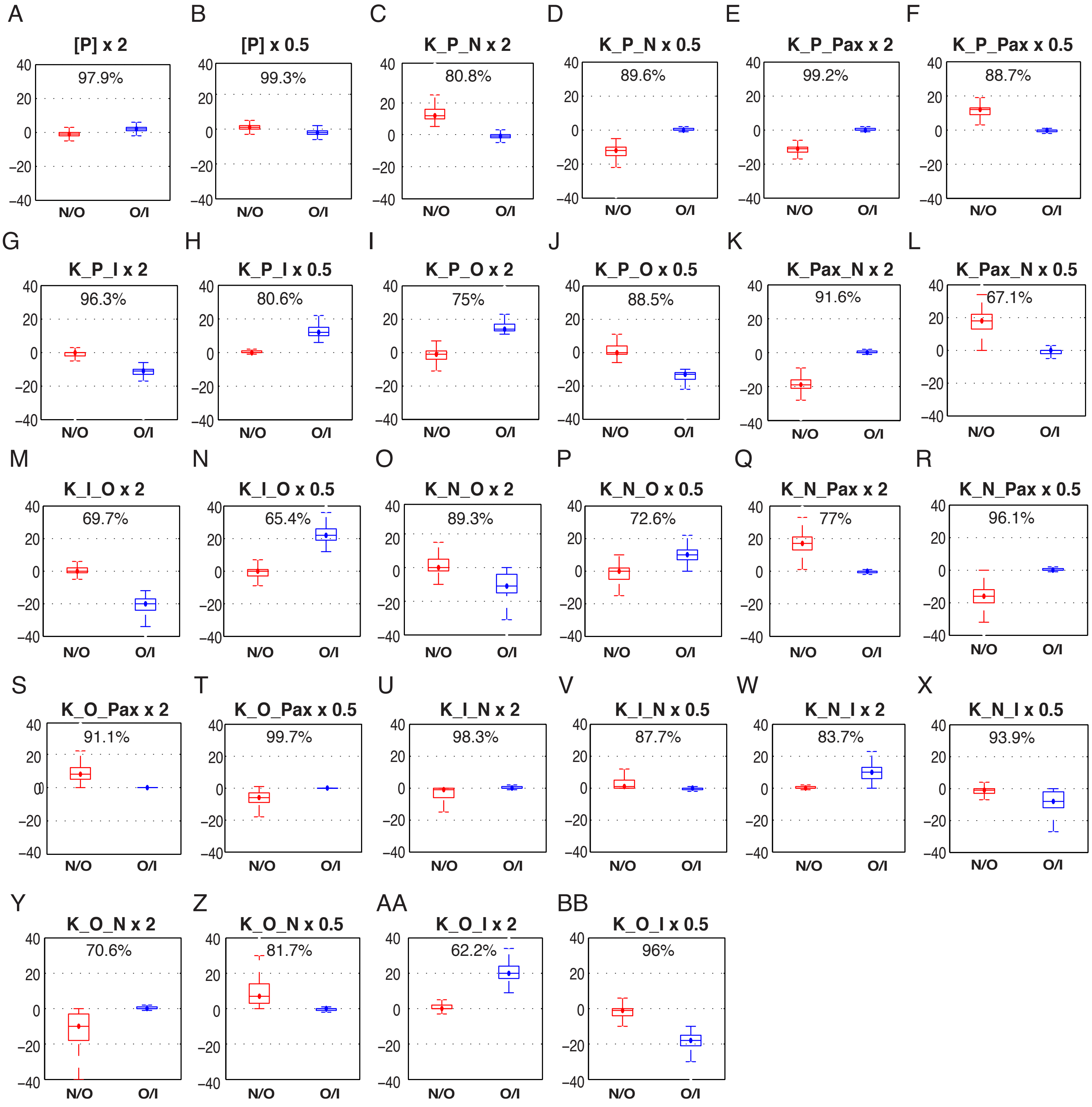
A
[P] x 2
97.9%
B
[P] x 0.5
99.3%
C
K_P_N x 2
80.8%
D
K_P_N x 0.5
89.6%
E
K_P_Pax x 2
99.2%
F
K_P_Pax x 0.5
88.7%
G
K_P_I x 2
96.3%
H
K_P_I x 0.5
80.6%
I
K_P_O x 2
75%
J
K_P_O x 0.5
88.5%
K
K_Pax_N x 2
91.6%
L
K_Pax_N x 0.5
67.1%
M
K_I_O x 2
69.7%
N
K_I_O x 0.5
65.4%
O
K_N_O x 2
89.3%
P
K_N_O x 0.5
72.6%
Q
K_N_Pax x 2
77%
R
K_N_Pax x 0.5
96.1%
S
K_O_Pax x 2
91.1%
T
K_O_Pax x 0.5
99.7%
U
K_I_N x 2
98.3%
V
K_I_N x 0.5
87.7%
W
K_N_I x 2
83.7%
X
K_N_I x 0.5
93.9%
Y
K_O_N x 2
70.6%
Z
K_O_N x 0.5
81.7%
AA
K_O_I x 2
62.2%
BB
K_O_I x 0.5
96%
40
20
0
−20
−40
N/O
O/I
